# Effect of including damage at the tissue level in the nonlinear homogenisation of trabecular bone

**DOI:** 10.1007/s10237-017-0913-7

**Published:** 2017-05-12

**Authors:** Francesc Levrero-Florencio, Krishnagoud Manda, Lee Margetts, Pankaj Pankaj

**Affiliations:** 1 School of Engineering, The University of Edinburgh, The King’s Buildings, Edinburgh, EH9 3DW UK; 2 School of Mechanical, Aerospace and Civil Engineering, The University of Manchester, Sackville Street, Manchester, M13 0PL UK

**Keywords:** Trabecular bone, Multiscale modelling, Plasticity, Damage, Finite element method

## Abstract

Being able to predict bone fracture or implant stability needs a proper constitutive model of trabecular bone at the macroscale in multiaxial, non-monotonic loading modes. Its macroscopic damage behaviour has been investigated experimentally in the past, mostly with the restriction of uniaxial cyclic loading experiments for different samples, which does not allow for the investigation of several load cases in the same sample as damage in one direction may affect the behaviour in other directions. Homogenised finite element models of whole bones have the potential to assess complicated scenarios and thus improve clinical predictions. The aim of this study is to use a homogenisation-based multiscale procedure to upscale the damage behaviour of bone from an assumed solid phase constitutive law and investigate its multiaxial behaviour for the first time. Twelve cubic specimens were each submitted to nine proportional strain histories by using a parallel code developed *in-house*. Evolution of post-elastic properties for trabecular bone was assessed for a small range of macroscopic plastic strains in these nine load cases. Damage evolution was found to be non-isotropic, and both damage and hardening were found to depend on the loading mode (tensile, compression or shear); both were characterised by linear laws with relatively high coefficients of determination. It is expected that the knowledge of the macroscopic behaviour of trabecular bone gained in this study will help in creating more precise continuum FE models of whole bones that improve clinical predictions.

## Introduction

Bone is a hierarchical biomaterial, exhibiting complex post-yield properties at each of its scales (Schwiedrzik et al. 2013). The mechanical behaviour of trabecular bone at the macroscale is often modelled by using homogeneous isotropic linear elasticity, with separate sets of elastic constants being assigned to cortical and trabecular bone (Completo et al. 2009; Conlisk et al. 2015). Site-specific mineral heterogeneity at the macroscale is often included by using computed tomography (CT) scans, which allow for variation of properties on the basis of CT attenuations (Helgason et al. 2008; Schileo et al. 2008; Tassani et al. 2011). However, bone mineral density alone is not enough to accurately predict the stiffness of trabecular bone at the macroscale since it is known to be anisotropic, mostly because of its heavily directional microstructure (Odgaard et al. 1997; Turner et al. 1990).

The macroscopic stiffness tensor of trabecular bone has been evaluated by using micro-CT ($\upmu$CT) and homogenisation-based multiscale finite element (FE) models, the so-called micro-FE ($\upmu$FE) models (Hollister et al. 1994; van Rietbergen et al. 1995). This method consists of picking a cubic region and converting the binarised $\upmu$CT scans to high resolution FE meshes, which include detailed geometry of bone microstructure. The solid phase is usually assigned isotropic elastic properties, and the cubic specimen is then subjected to six virtual load cases, stress or strain (Hollister and Kikuchi 1992). The response from these tests enables the evaluation of the macroscopic elastic stiffness tensor by using a standard mechanics approach (van Rietbergen et al. 1996). This methodology has been extensively used, and studies have also established relationships between these stiffness tensors and the micro-architectural indices of the considered samples, specifically bone volume over total volume fraction (BV/TV) and fabric tensor (Cowin 1985; Zysset and Curnier 1995; Zysset 2003).

This approach has been extended to predict the yield criterion of trabecular bone (Cowin 1986; Bayraktar and Keaveny 2004; Wolfram et al. 2012; Sanyal et al. 2015; Levrero-Florencio et al. 2016). These studies use FE meshes derived from $\upmu$CT scans which are subjected to multiple load cases to evaluate the homogenised yield surface. The 0.2% criterion is then used to assess where the yield point is located, which means that the elastic slope intercepts the *X*-axis at a value of 0.2% macroscopic strain. In a multiaxial context, the macroscopic yield point is located where the macroscopic elastic slope intercepts the macroscopic stress norm−macroscopic strain norm curve. Isotropic elastoplastic assumption is made for the constitutive law at the solid phase level. Some of these studies used an asymmetric principal strain-based criterion to represent the onset of yield (Bayraktar and Keaveny 2004; Wolfram et al. 2012; Sanyal et al. 2012), but others used an approximation to a Drucker–Prager yield surface (Panyasantisuk et al. 2015; Levrero-Florencio et al. 2016), as suggested by Tai et al. (2006) and Carnelli et al. (2010).

Nanoindentation experiments on bone suggest that its solid phase can be effectively modelled by using a pressure-dependent yield surface, arising from its cohesive-frictional behaviour (Tai et al. 2006). Therefore, it has been suggested that the solid phase of bone can be modelled using classical yield surfaces such as Mohr–Coulomb or Drucker–Prager (Carnelli et al. 2010; Tai et al. 2006). The resulting macroscopic yield criteria have been defined using both stress and strain-based descriptions (Keaveny et al. 1994; Keller 1994; Kopperdahl and Keaveny 1998). Studies have shown that the yield surface in strain space is approximately isotropic and independent of BV/TV (Bayraktar and Keaveny 2004; Levrero-Florencio et al. 2016) and consequently easy to apply in macroscopic models (Pankaj 2013; Pankaj and Donaldson 2013). Maghous et al. (2009) showed that the macroscopic yield surface of a porous material with a Drucker–Prager yield surface for the matrix material reduces to an eccentric ellipsoid, with the corresponding decrease in uniaxial strength values due to the presence of porosity. This suggests that trabecular bone at the macroscale can be modelled with an eccentric ellipsoid, or Tsai–Wu, which has been shown by Cowin (1986), Wolfram et al. (2012), Panyasantisuk et al. (2015) and Levrero-Florencio et al. (2016).

After yield, there is little information on how the macroscopic response of trabecular bone evolves with further loading; hardening is usually assumed to be isotropic in computational models (Garcia et al. 2009; Schwiedrzik and Zysset 2013). It is not possible to experimentally test different load directions after yield in the same sample since samples tested once cannot be retested as damage in one direction may affect the rest of directions and finding two or more samples with highly resembling microstructure is not possible. This makes it impossible to experimentally obtain the macroscopic multiaxial post-yield behaviour of trabecular bone. The $\upmu$FE approach again presents an opportunity to understand this via computational means. To evaluate the macroscopic post-yield response, once again, the solid phase constitutive model needs to be provided. Bone shows two main mechanisms of energy dissipation after yield: plastic deformation and elastic stiffness reduction, or damage (Schwiedrzik and Zysset 2013). With regard to hardening of the solid phase, it has been assumed to be linear, with a slope of 5% its elastic stiffness, in previous homogenisation studies (Bayraktar and Keaveny 2004; Wolfram et al. 2012). A recent study showed that the hardening of the extracellular matrix, which can be considered to be a scale below the solid phase of trabecular bone, is, however, slightly nonlinear (Schwiedrzik et al. 2014).

Damage behaviour of bone at different scales has been studied and modelled in several studies (Keaveny et al. 1999; Schwiedrzik and Zysset 2013; Garcia et al. 2009). Garcia et al. (2009) developed a macroscopic constitutive model for bone—a yield surface defined in stress space using the fabric-based elastic compliance tensor and a damage threshold modelled with a halfspacewise generalisation of the Hill criterion (Rincón-Kohli and Zysset 2009). Schwiedrzik and Zysset (2013) developed a constitutive model for bone which is potentially applicable to different length scales, ranging from the ultrastructural to the macroscopic level. It includes anisotropic elasticity based on a multiscale homogenisation scheme proposed by Reisinger et al. (2010), an eccentric elliptical surface which describes the onset of yield and damage (Wolfram et al. 2012; Levrero-Florencio et al. 2016), and viscoplasticity described by a Perzyna formulation (Ponthot 1998). The damage variable used in these two above-cited studies is scalar and thus describes isotropic damage evolution, i.e. damage equally affects all directions. While this presents a relatively simple model, it appears unlikely that damage due to loading in one direction will affect stiffness components, isotropically, in all directions.

This study uses a $\upmu$FE-based nonlinear homogenisation approach, with plasticity and damage, to evaluate how assumptions made for the solid phase of trabecular bone affect its macroscopic post-yield behaviour. The first aim of this study is to assess how the macroscopic stiffness components are affected by the initiation and development of microscopic damage. The second aim is to assess how macroscopic damage is related to the macroscopic strain norm and how well it can be predicted for different load cases. The third aim is to assess the evolution of the macroscopic yield surface, in both strain and stress space.

## Materials and methods

### Experimental samples and imaging procedure

Fresh bovine proximal femurs (${<}2.5$ years old) were obtained from a local abattoir and were stored at $-20\,^{\circ }\hbox {C}$ until they were cored. Ten cores were extracted from the trochanteric region of these femurs. Diamond-coated coring tools (Starlite Industries, Rosemont PA, USA) were used to extract the specimens, and then the top and bottom faces of the cylinders were cut with a slow-speed saw (Isomet 1000, Buehler, Düsseldorf, Germany). $\upmu$CT scans were taken for each specimen by using a Skyscan 1172 $\upmu$CT scanner (Bruker, Kontich, Belgium) at a resolution of 17.22 $\upmu$m. The scanning parameters were 94 kV, 136 mA and 200 ms integration time with four scans taken in 720 equiangular positions. The grey-scale images were binarised with a thresholding script that does not require user intervention (Gómez et al. 2013).

Twelve 5-mm virtual cubes were extracted from the binarised cylinders. This volume element (VE) size has been previously used and therefore is considered to be appropriate to capture the features of trabecular bone (Harrigan et al. 1988; van Rietbergen et al. 1995; Sanyal et al. 2015). The mean intercept length (MIL) fabric tensor (Harrigan and Mann 1984) was evaluated using BoneJ (Doube et al. 2010) and then used to align the images with the fabric. This approach has been used in previous studies (Wolfram et al. 2012; Levrero-Florencio et al. 2016). The alignment of the cubes was rechecked after the 5 mm cropping to ensure that the misalignment was smaller than $8^{\circ }$ (Wolfram et al. 2012; Sanyal et al. 2015; Levrero-Florencio et al. 2016). The BV/TV of the samples ranged from 14.8 to 30.3%, and their degree of anisotropy (DOA) ranged from 1.61 to 3.47.

### Solid phase constitutive model

The mathematical operators defined in this section largely follow the notation used in Schwiedrzik et al. (2013), Panyasantisuk et al. (2015) and Levrero-Florencio et al. (2016). We have used tensor notation, which can be alternatively expressed using index notation as shown within the parentheses in the following. For example, a second-order tensor and its representation in index notation are: ${\mathbf{A }} ({{{A}}}_{ij})$. A double contraction of a second-order tensor and a fourth-order tensor is expressed as $\mathbf{A }:{\mathbb {B}} ({{A}}_{ij}{\mathbb {B}}_{ijkl})$; a double contraction of a fourth-order tensor and a second-order tensor is expressed as ${\mathbb {A}}:\mathbf{B } ({\mathbb {A}}_{ijkl}{{B}}_{kl})$; a double contraction of two second-order tensors is expressed as $\mathbf{A }:\mathbf{B } ({{A}}_{ij}{{B}}_{ij})$; a tensor product between two second-order tensors is expressed as $\mathbf{A }\otimes \mathbf{B } ({{A}}_{ij}{{B}}_{kl})$; and a symmetric tensor product between two second-order tensors is expressed as $\mathbf{A }\,\underline{\overline{\otimes }}\,\mathbf{B } (\frac{1}{2}[{{A}}_{ik}{{B}}_{jl}+{{A}}_{il}{{B}}_{jk}])$.

The elastic regime of the solid phase was defined as an isotropic linear material, by using Hencky’s hyperelasticity (de Souza Neto et al. 2008), with a Young’s Modulus of 12,700 MPa and a Poisson’s ratio of 0.3 (Wolfram et al. 2012; Levrero-Florencio et al. 2016). As Cowin (1997) stated, there is little to no error in assuming tissue isotropy for trabecular bone. A quadric yield surface was used to describe the onset of yield and damage of the solid phase (Schwiedrzik et al. 2013; Schwiedrzik and Zysset 2015). The yield function *f* was defined as

1 $$\begin{aligned} f(\varvec{\sigma })=\sqrt{\varvec{\sigma }:{\mathbb {F}}:\varvec{\sigma }}+\mathbf{F }:\varvec{\sigma }-R(\overline{\varepsilon }^{p})=0, \end{aligned}$$

where $\varvec{\sigma }$ is the stress, ${\mathbb {F}}$ and $\mathbf{F }$ are, respectively, fourth- and second-order tensors which define the shape and eccentricity of the yield surface, *R* is the radius of the isotropic yield criterion, and $\overline{\varepsilon }^{p}$ is the accumulated plastic strain. Note that the yield surface is defined in stress space, and not in effective stress space (Schwiedrzik and Zysset 2015).

The fourth-order tensor ${\mathbb {F}}$ and the second-order tensor $\mathbf{F}$ are defined as

2 $$\begin{aligned} {\mathbb {F}}=-\zeta _0F_0^2(\mathbf{I }\,\otimes \,\mathbf{I })+(\zeta _0+1)F_0^2(\mathbf{I }\,\underline{\overline{\otimes }}\,\mathbf I ) \end{aligned}$$

and

3 $$\begin{aligned} \mathbf{F }=\frac{1}{2}\left( \frac{1}{\sigma _0^+}-\frac{1}{\sigma _0^-}\right) \mathbf I , \end{aligned}$$

where

4 $$\begin{aligned} F_0=\frac{\sigma _0^++\sigma _0^-}{2\sigma _0^+\sigma _0^-}. \end{aligned}$$

Equation 1 approximates a Drucker–Prager criterion when $\zeta _{0}$ = 0.49. Recent nanoindentation studies on bone tissue suggest that a Mohr–Coulomb or a Drucker–Prager surface could approximate the yield criterion at the microscopic level (Tai et al. 2006; Carnelli et al. 2010) and it has been recently used in homogenisation studies (Panyasantisuk et al. 2015; Levrero-Florencio et al. 2016). The uniaxial yield strains for use in the criterion were assumed to be 0.41% in tension and 0.83% in compression (Bayraktar and Keaveny 2004) and were then converted to yield stresses by using the procedure described in Schwiedrzik et al. (2016). This means that the corresponding yield stresses are $E_0\,\varepsilon _0^+=52$ MPa for tension and $E_0\,\varepsilon _0^-=105$ MPa for compression, where $E_0$ is the undamaged Young’s modulus. Linear isotropic hardening of 5% of the elastic slope was also assumed (Wolfram et al. 2012; Sanyal et al. 2015; Panyasantisuk et al. 2015).

Isotropic damage evolution was assumed to be coupled with plasticity, exactly as in Schwiedrzik and Zysset (2013, 2015), and thus it is defined as

5 $$\begin{aligned} D(\overline{\varepsilon }^{p})=D_{c}(1-\text {e}^{-k_{p}\overline{\varepsilon }^{p}}), \end{aligned}$$

where $D_{c}$ is the maximum damage, which is capped at 0.9 to avoid numerical difficulties related to the complete loss of stress carrying capacity in any region of the model; the inverse damage rate $1/k_p$ was set to 9.53% (Schwiedrzik and Zysset 2013). Implementation of the model in an implicit FE context is described in “Appendix”.

### Computational procedure

The twelve VEs were meshed with trilinear hexahedra, by converting every voxel of the binarised CT scans to a FE element. The largest obtained mesh had around nine million nodes and thus around 27 million degrees of freedom.

Each of the VEs was subjected to nine strain-controlled uniaxial cases: three tensile, three compressive and three shear cases. Kinematic uniform boundary conditions were used to constrain the VEs, which were applied as described by Wang et al. (2009). According to Wang et al. (2009) and Panyasantisuk et al. (2015), these boundary conditions (BC) provide an upper bound for the macroscopic stiffness tensor and macroscopic yield surface of trabecular bone.

FE simulations were run on a Cray XC30 supercomputer hosted by ARCHER (UK National Supercomputing Service). The used software was an *in-house* parallel implicit finite strain FE solver, developed within the context of ParaFEM (Margetts 2002; Smith et al. 2013), which uses Message Passing Interface (MPI) to perform the parallelisation (The MPI Forum 1993). Each simulation took from 40 to 100 min when using 1920 cores, depending on the load case, with compression load cases taking the longest. The initial step size corresponded to 0.1% macroscopic strain norm and was permitted to decrease to a minimum of 0.001% if global or local convergence was not achieved in larger increments.

**Fig. 1:**
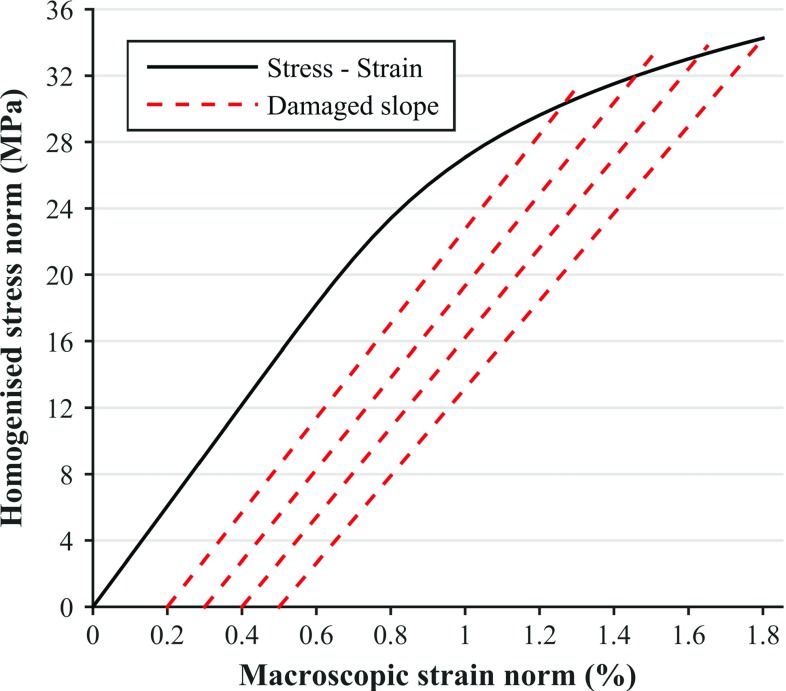
Definition of the macroscopic strain points with the damaged slopes; this is the compression case in direction 1 (i.e. $-\varepsilon _{11}$) for the densest sample ($\hbox {BV}/\hbox {TV}=30.3\%$). The slope at 0.5% macroscopic strain norm is approximately 12% lower than the undamaged slope

In order to enlarge the region of convergence of the Newton-Closest-Point Projection Method (Newton-CPPM) scheme, a line search procedure was implemented as in the primal-CPPM algorithm proposed by Pérez-Foguet and Armero (2002). To ensure that a possible fail of convergence of the CPPM scheme does not influence the results of our FE simulation, lack of convergence of the CPPM at any integration point is broadcasted to all MPI processes in order to cut down the time increment to half of its value. A Newton-Raphson scheme was used as the global solution tracking procedure, and a preconditioned conjugate gradient method was used as the linear algebraic solver.

### Definition of the macroscopic strain points

The yield points were described in the homogenised Green-Lagrange strain norm−homogenised Second Piola–Kirchhoff stress norm plane (Fig. 1). The homogenised stress is then defined as

6 $$\begin{aligned} \varvec{\sigma }_\mathrm{hom}=\frac{1}{V_{0}}\sum _{i=1}^\mathrm{nel}\sum _{j=1}^\mathrm{nip}w_{i}\text {det}(\mathbf {J}_{\textit{ij}})\,\varvec{\sigma }_{\textit{ij}}, \end{aligned}$$

where no summation is implied over repeated indices, $V_{0}$ is the initial volume of the VE, nel is the number of elements in the system, nip is the number of integration points in a trilinear hexahedron, $w_{i}$ are the weights corresponding to a trilinear hexahedron, $\mathbf{J }$ is the Jacobian, and $\varvec{\sigma }$ is the stress at the solid phase level. Note that due to the relatively small yield strains, an infinitesimal strain formulation can be used at the macroscale (Wolfram et al. 2012; Schwiedrzik et al. 2014).

The homogenised orthotropic elastic stiffness tensor was calculated at every time increment using the procedure described by van Rietbergen et al. (1995, 1996), and the damage variable was used to reduce the integration point-specific solid phase stiffness tensor. Since the sample was already aligned according to the directions described by the MIL fabric tensor, the resulting elasticity tensor was assumed to be orthotropic and aligned with the MIL axes (Odgaard et al. 1997). The 0.2% criterion was used to define the yield points (Wolfram et al. 2012) and extended to further define additional strain points at 0.3, 0.4 and 0.5% by using the procedure shown in Fig. 1. These points will henceforth be referred to as macroscopic strain norms (e.g. 0.2% macroscopic strain norm). Note that if yield is considered to occur at 0.2%, the following points (0.3, 0.4 and 0.5%) could be considered as 0.1, 0.2 and 0.3% macroscopic plastic strain norms, respectively (with damaged slope). Clearly, these points defined with the damage will correspond to larger macroscopic total strains in comparison with those evaluated without damage. The appropriate damaged slope to define the strain points is calculated for the corresponding load case at each time step, by using the damaged macroscopic stiffness tensor. An example is presented here for a strain-controlled uniaxial case in direction 1.

In the following, the homogenised stress is the projection of the macroscopic elastic strain through the macroscopic damaged stiffness tensor, the first subscript denotes a label, and the following subscripts denote indices of the tensor. Consider application of a normal strain in direction 1, the elastic system can be written in indicial notation as

7 $$\begin{aligned} \sigma _{\mathrm{hom},ij}=D_{\mathrm{dam}, ij11}\epsilon _{0,11}^e, \end{aligned}$$

where $D_{\mathrm{dam},ijkl}$ are the components of the damaged macroscopic stiffness tensor. When the norm of the corresponding homogenised stress is calculated, the following expression can be derived, by taking into account the orthotropy of the macroscopic stiffness tensor,

8 $$\begin{aligned} \begin{aligned}&\Vert D_{\mathrm{dam},ij11}\epsilon _{0,11}^e\Vert \\&\quad =\sqrt{D_{\mathrm{dam},1111}^2\epsilon _{0,11}^{e,2}+D_{\mathrm{dam},2211}^2\epsilon _{0,11}^{e,2}+D_{\mathrm{dam},3311}^2\epsilon _{0,11}^{e,2}}, \end{aligned} \end{aligned}$$

and thus the damaged slope used to calculate the macroscopic strain points can be expressed as

9 $$\begin{aligned} K_{\mathrm{dam}}=\sqrt{(D_{\mathrm{dam},1111}^2+D_{\mathrm{dam},2211}^2+D_{\mathrm{dam},3311}^2)}. \end{aligned}$$

## Results

### Stiffness reduction

**Fig. 2:**
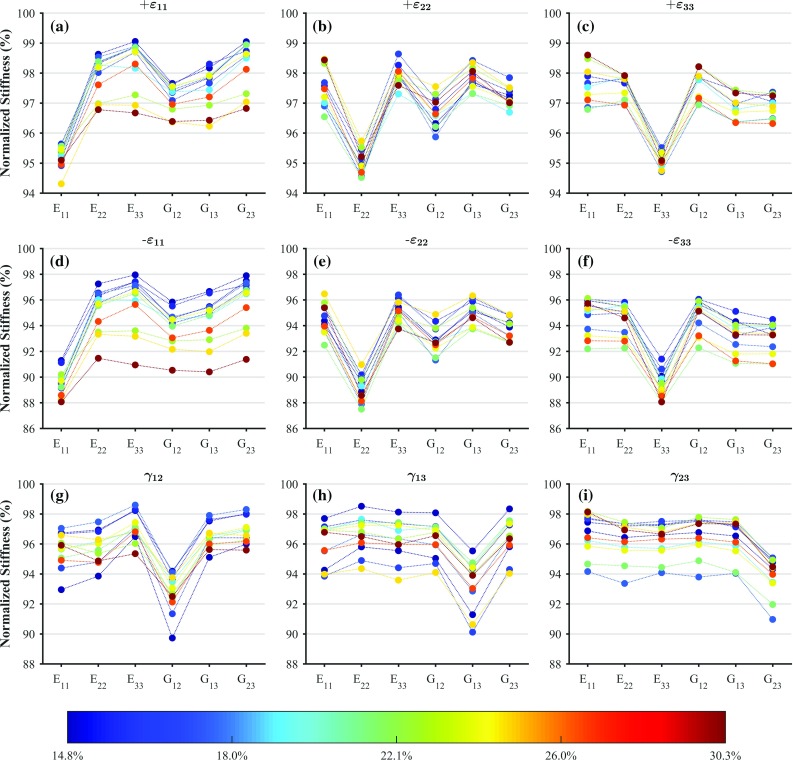
Normalised orthotropic stiffness for all the samples, and for all the considered strain-controlled uniaxial load cases: tensile loading in direction 1(**a**), 2(**b**) and 3(**c**) ($+\varepsilon _{11}$, $+\varepsilon _{22}$ and $+\varepsilon _{33}$, respectively); compressive loading in direction 1(**d**), 2(**e**) and 3(**f**) ($-\varepsilon _{11}$, $-\varepsilon _{22}$ and $-\varepsilon _{33}$, respectively); and shear loading in plane 1–2(**g**), 1–3(**h**) and 2–3(**i**) ($\gamma _{12}$, $\gamma _{13}$ and $\gamma _{23}$, respectively). The colour coding is on the basis of BV/TV and is used as a labelling mechanism. The points defined at 0.5% macroscopic strain norm have been considered in this figure

The damaged orthotropic stiffness components ($E_{11}$, $E_{22}$, $E_{33}$, $G_{12}$, $G_{13}$ and $G_{23}$) are obtained from the damaged macroscopic stiffness tensor. These are then normalised by dividing them by the corresponding undamaged orthotropic stiffness and plotted for every sample and for every considered load case (Fig. 2). Figure 2 shows that in spite of isotropic damage being assumed at the solid phase level, its effect on the macroscopic level is not isotropic, and that its effect depends on the considered loading mode (i.e. tension, compression or shear). It can be seen that while all stiffness components reduce in all load cases, the stiffness component corresponding to the load case the sample is subjected to reduces the most. It is also interesting to note that in the case of a strain-controlled uniaxial normal load case, the shear stiffness components corresponding to the shear planes containing the loaded normal component reduce more than the other one (e.g. if the normal case is in direction 1, $G_{12}$ and $G_{13}$ reduce more than $G_{23}$).

**Fig. 3:**
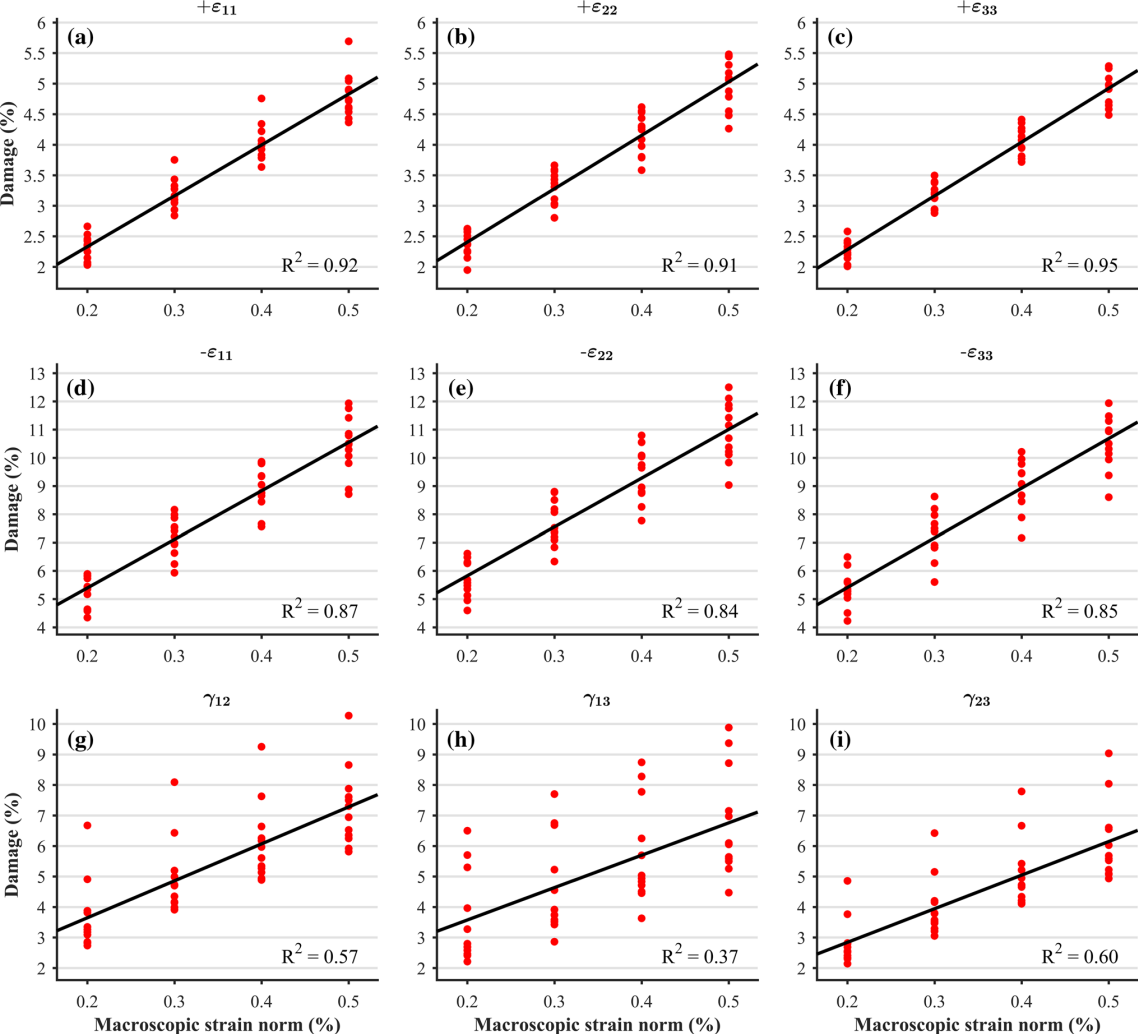
Linear fits between damage and macroscopic strain norms, and the corresponding data points. The considered uniaxial strain-controlled load cases are: tensile loading in direction 1(**a**), 2(**b**) and 3(**c**) ($+\varepsilon _{11}$, $+\varepsilon _{22}$ and $+\varepsilon _{33}$, respectively); compressive loading in direction 1(**d**), 2(**e**) and 3(**f**) ($-\varepsilon _{11}$, $-\varepsilon _{22}$ and $-\varepsilon _{33}$, respectively); and shear loading in plane 1–2(**g**), 1–3(**h**) and 2–3(**i**) ($\gamma _{12}$, $\gamma _{13}$ and $\gamma _{23}$, respectively)

Stiffness reductions for each of the considered load cases were related to BV/TV, corresponding initial orthotropic stiffness component, and macroscopic strain norm through multilinear regression analyses. Only relationships with respect to macroscopic strain norms were found to be significant ($p<0.05$). Therefore, we re-evaluated these multilinear regressions as linear regressions, only with respect to macroscopic strain norms ($p\rightarrow 0$). The coefficients of determination ($R^2$), the intercepts and the slopes of these fits are shown in Table 1. Figure 3 illustrates these fits along with the actual data points. It can be seen from this figure that damage development under strain-controlled uniaxial tension and strain-controlled uniaxial compression can be reasonably well predicted by a linear relationship with respect to the macroscopic strain norm, but not so well for shear, as the coefficients of determination suggest. It is also important to point out that for the considered range of post-elastic strains, damage development can be reasonably well approximated with a line although the relationship between damage and accumulated plastic strain at the solid phase is exponential (Eq. 5).

**Table 1:** Values of the coefficients of determination $R^2$, intercepts and slopes for the linear fits between damage and macroscopic strain norms, for each of the considered load cases

Load case	$+\epsilon _{11}$	$+\epsilon _{22}$	$+\epsilon _{33}$	$-\epsilon _{11}$	$-\epsilon _{22}$	$-\epsilon _{33}$	$\gamma _{12}$	$\gamma _{13}$	$\gamma _{23}$
${R}^2$	0.92	0.91	0.95	0.87	0.84	0.85	0.57	0.37	0.60
Intercept (%)	0.66	0.66	0.52	1.96	2.37	1.89	1.22	1.45	0.64
Slope (%)	832.7	874.4	880.0	1717.5	1726.6	1757.6	1211.3	1061.6	1101.5

**Fig. 4:**
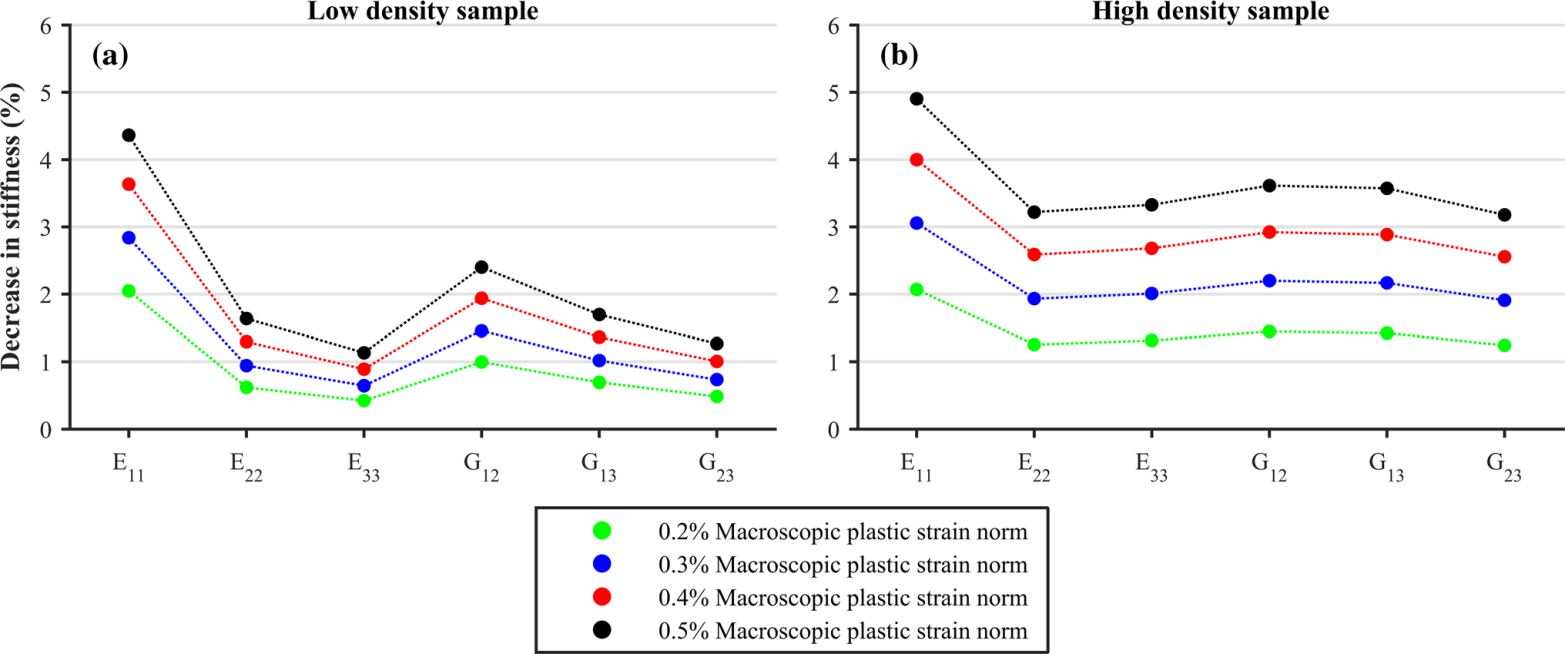
Decrease in stiffness components for the most porous ($\hbox {BV}/\hbox {TV}=14.8\%$) and densest ($\hbox {BV}/\hbox {TV}=30.3\%$) samples due to tensile loading in direction 1 (i.e. $+\varepsilon _{11}$), and for all the considered macroscopic strain norm levels

Figure 4 shows the stiffness reduction for the most porous and the densest samples, for the load case in which a strain-controlled uniaxial tension is applied in direction 1, for different macroscopic strain norm levels. As expected, the decrease in stiffness increases with increasing strain norm, a trend which was true for all the samples. For the samples shown, it can also be seen that in the denser sample the difference in stiffness reduction between the stiffness component corresponding to the load case the sample is subjected to and the others is smaller than for the porous sample. Therefore, we considered linear relationship between BV/TV and the difference between the stiffness reduction in the component corresponding to the load case the sample is subjected to and the average of the rest of stiffness components. However, poor statistical significance was found ($p>0.05$), indicating that this was not a general trend.

### Hardening of the macroscopic yield surface

**Fig. 5:**
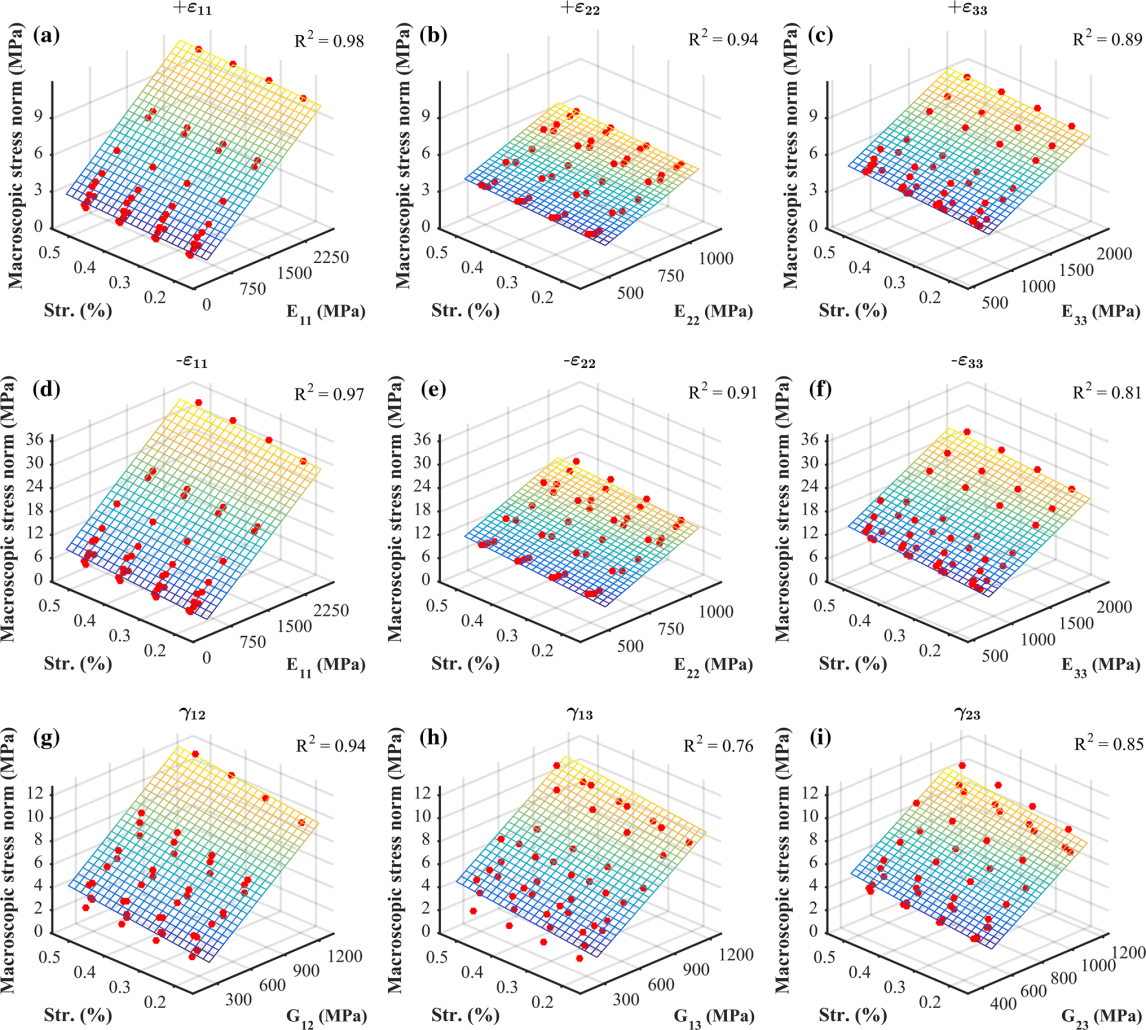
Macroscopic yield stress norms for each of the considered load cases, for all the considered samples and for all the considered macroscopic strain norms (Str. stands for macroscopic strain norm). The considered uniaxial strain-controlled load cases are: tensile loading in direction 1(**a**), 2(**b**) and 3(**c**) ($+\varepsilon _{11}$, $+\varepsilon _{22}$ and $+\varepsilon _{33}$, respectively); compressive loading in direction 1(**d**), 2(**e**) and 3(**f**) ($-\varepsilon _{11}$, $-\varepsilon _{22}$ and $-\varepsilon _{33}$, respectively); and shear loading in plane 1–2(**g**), 1–3(**h**) and 2–3(**i**) ($\gamma _{12}$, $\gamma _{13}$ and $\gamma _{23}$, respectively)

Macroscopic yield stresses have been related to fabric and BV/TV in previous studies; however, relationships between macroscopic yield strains and BV/TV are weak and relationships between macroscopic yield strains and fabric are moderate, but significant (Wolfram et al. 2012; Panyasantisuk et al. 2015). Also, as previously stated, the orthotropic stiffness components have been related to these micro-architectural indices (Odgaard et al. 1997; Zysset 2003). Therefore, we considered inclusion of orthotropic stiffness components in our regressions for the yield stresses (rather than yield strains). Macroscopic yield stress norms were related to the corresponding initial orthotropic stiffness and the macroscopic strain norms through multilinear regressions. These fits and the corresponding data points are shown in Fig. 5. It can be seen that higher yield stress results from higher initial stiffness and from the choice of higher macroscopic strain norm. The coefficients of determination and slopes of these fits are shown in Table 2. All of these fits were found to be statistically significant ($p<0.05$).

**Fig. 6:**
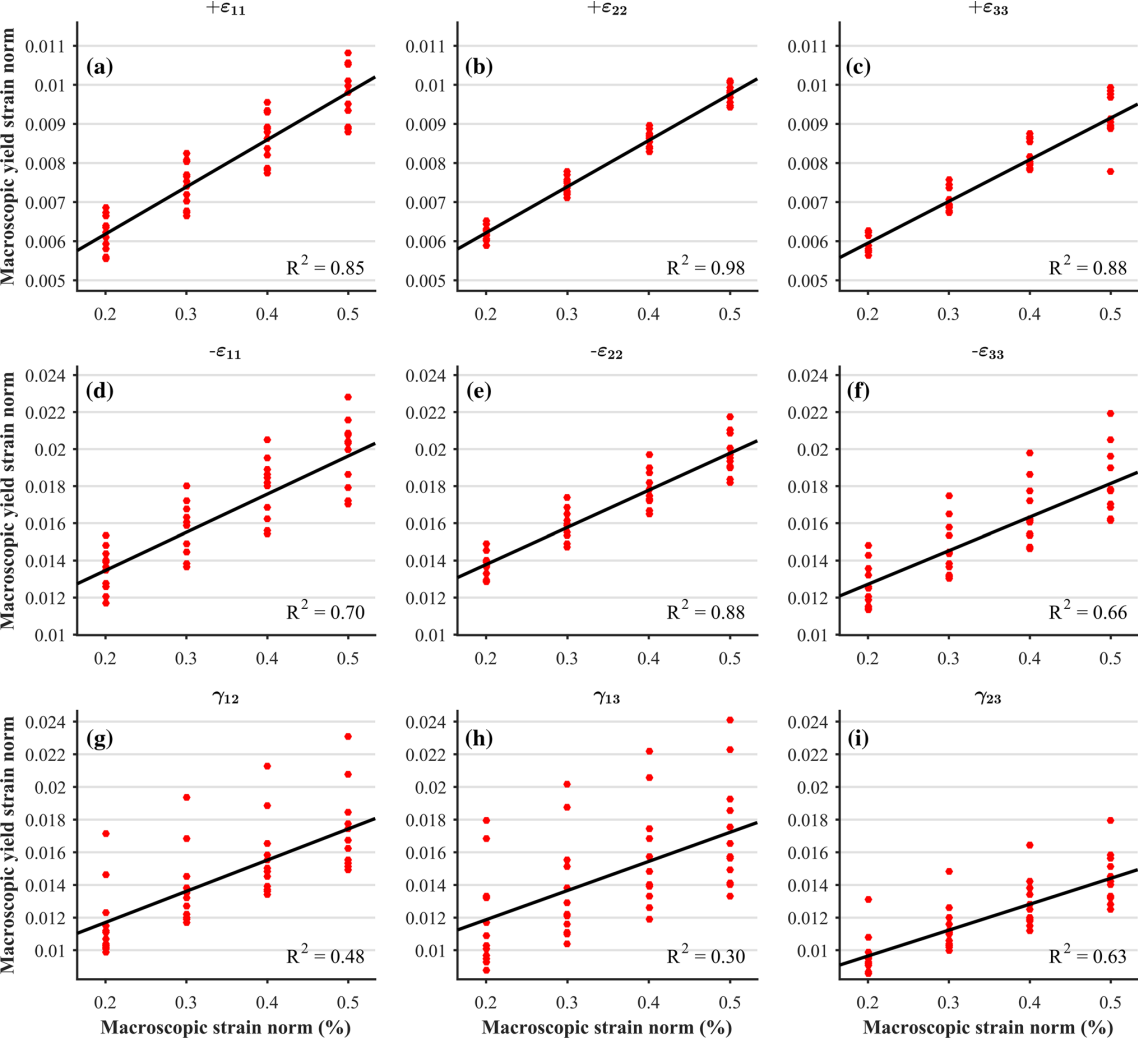
Macroscopic yield strains for each of the considered load cases, for all the considered samples and for all the considered macroscopic strain norms. The considered uniaxial strain-controlled load cases are: tensile loading in direction 1(**a**), 2(**b**) and 3(**c**) ($+\varepsilon _{11}$, $+\varepsilon _{22}$ and $+\varepsilon _{33}$, respectively); compressive loading in direction 1(**d**), 2(**e**) and 3(**f**) ($-\varepsilon _{11}$, $-\varepsilon _{22}$ and $-\varepsilon _{33}$, respectively); and shear loading in plane 1–2(**g**), 1–3(**h**) and 2–3(**i**) ($\gamma _{12}$, $\gamma _{13}$ and $\gamma _{23}$, respectively)

Macroscopic yield strain norms were related to the macroscopic strain norms through linear regressions. These fits and the corresponding data points are shown in Fig. 6. The coefficients of determination and slopes of these fits are provided in Table 3. All of these fits were statistically significant ($p<0.05$). In general, it is found that hardening in both stress and strain spaces depends on the loading mode, i.e. tension, compression or shear, but it is not anisotropic.

## Discussion

Damage behaviour of trabecular bone at the macroscale has been assessed in some previous studies for relatively simple load scenarios (Zioupos et al. 2008; Garcia et al. 2009; Sun et al. 2010). Damage at the microscopic level has also been previously considered (Gupta et al. 2006). This study aims to bridge both scales by investigating the damage behaviour of trabecular bone at the macroscale through a homogenisation-based multiscale approach and the use of multiple load cases for trabecular bone samples with very detailed geometry. Twelve $\upmu$FE meshes of samples covering a wide range of BV/TV and nine strain-controlled uniaxial load cases per sample were investigated with plasticity and damage included at the solid phase level.

The constitutive behaviour of the solid phase, including its damage behaviour, was considered to be isotropic. Cowin (1997) stated that the assumption of isotropy leads to little to no error. This is because trabeculae are composed of laminated material about their axes, which implies transverse isotropy or orthotropy; since the axis of a trabecula is the same as its loading axis, a beam made of orthotropic material can be reduced to a beam made of isotropic material. However, isotropic damage at the microscale results in anisotropic macroscale damage response, and depending on the loading scenario (Fig. 2). It is also interesting to note that strain-controlled uniaxial compressive or tensile loading results in damage not only in the direction of loading but also in other normal and shear directions. Shi et al. (2010) suggested that tissue yielding and microdamage only have a moderately strong correlation, whereas our tissue constitutive model directly links damage and yielding, as damage explicitly depends on the accumulated plastic strain. Nonetheless, they also suggested that there is a larger proportion of damaged tissue in the longitudinal trabeculae (direction of loading), which is in agreement with our results as the most damaged orthotropic stiffness component is always the on-axis component. Some previous studies which have modelled damage at the macroscale have assumed an isotropic behaviour (Schwiedrzik and Zysset 2013; Garcia et al. 2009), which may be an acceptable assumption for proportional loading, but not for changing loads, as would be expected during physiological activities.

Damage (Fig. 3) can be linearly related to the macroscopic strain norm with high coefficients of determination ($R^2>0.84$), except for shear cases ($R^2<0.60$). This also suggests that the evolution of damage at the macroscale can be assumed to be linear in the range of considered macroscopic strain norms (0.2–0.5%). Beyond these strain levels, other effects, such as cracking and fracture of trabecula, can lead to structural failure and softening if further loading is applied (Kopperdahl and Keaveny 1998; Hosseini et al. 2014); this was not considered in this study since it is expected that the relatively low levels of macroscopic strain will not trigger these effects.

**Table 2:** Values of the coefficient of determination $R^2$ and slopes for the linear fits between macroscopic yield stress norms, corresponding initial stiffness and macroscopic strain norms, for each of the considered load cases

Load case	$+\epsilon _{11}$	$+\epsilon _{22}$	$+\epsilon _{33}$	$-\epsilon _{11}$	$-\epsilon _{22}$	$-\epsilon _{33}$	$\gamma _{12}$	$\gamma _{13}$	$\gamma _{23}$
${R}^2$	0.98	0.94	0.90	0.97	0.91	0.81	0.94	0.77	0.85
Slope with respect to initial stiffness	3.73E−3	5.19E−3	3.40E−3	11.31E−3	17.44E−3	10.83E−3	8.29E−3	7.34E−3	8.94E−3
Slope with respect to macroscopic strain norm (MPa)	121.85	135.48	175.69	648.97	679.47	687.42	362.56	344.11	339.19

**Table 3:** Values of the coefficient of determination $R^2$ and slopes for the linear fits between macroscopic yield strain norms and macroscopic strain norms, for each of the considered load cases

Load case	$+\epsilon _{11}$	$+\epsilon _{22}$	$+\epsilon _{33}$	$-\epsilon _{11}$	$-\epsilon _{22}$	$-\epsilon _{33}$	$\gamma _{12}$	$\gamma _{13}$	$\gamma _{23}$
$R^2$	0.85	0.98	0.88	0.70	0.88	0.65	0.48	0.30	0.63
Slope with respect to macroscopic strain norm	1.21	1.18	1.07	2.06	2.00	1.81	1.91	1.79	1.59

The values of the slopes of the linear fits are shown in Table 1; they show that damage propagation increases differently for strain-controlled uniaxial tension and compression load cases, with the value for compression cases being around twice the value for tension cases. This may be due to the fact that under compression, heterogeneous stress distributions occur that include tensile stresses at the solid phase level due to bending and buckling of trabeculae (Stölken and Kinney 2003; Bevill et al. 2006). Additionally, damage and plasticity in compression are far more diffused than in tension where they are more localised (Lambers et al. 2014). These lead to larger volumes of bone yielding (and thus being damaged as well) throughout the compression process when compared to tension. This is captured by the homogenisation procedure and expressed as a higher slope for the damage progression. In tension, cracks are more localised and propagate faster, eventually leading to catastrophic failure of individual trabeculae. Moreover, it is also likely that cracks under compression exhibit some partial closure since bone is a quasi-brittle material, leading to reduced effects of damage on the stiffness. Nonetheless, these effects have not been included in the solid phase constitutive model.

If individual samples are considered (Fig. 4), it can be seen that damage evolves with increasing macroscopic strain norm and that the low BV/TV sample has a considerable difference in damage between the stiffness component corresponding to the load case the sample is subjected to and the rest of stiffness components, effect which is not observed in the high BV/TV sample. However, the expectation that high BV/TV, more continuum-like, trabecular bone samples would partially upscale the isotropic damage behaviour of the solid phase was not supported by the statistical analysis as BV/TV was not found to be a good predictor of this. Perhaps, more samples with a wider range of BV/TV and rod/plate morphology could lead to morphological parameters being related to damage.

Several previous studies (Wolfram et al. 2012; Levrero-Florencio et al. 2016; Panyasantisuk et al. 2015) used the 0.2% criterion to determine the macroscopic yield of trabecular bone. However, this study shows that if damage is included, it results in a certain reduction of stiffness at the 0.2% macroscopic strain norm (Table 1). This implies that a modified elasticity tensor may need to be used once macroscopic yield is encountered. This can be done by considering a damaged slope obtained by joining the origin and the yield stress at 0.2% macroscopic strain norm. Previous studies have employed an isotropic reduction of the elastic stiffness (Wolfram et al. 2012); however, this study shows that the macroscopic damage may not be isotropic.

With respect to the hardening of trabecular bone at the macroscale, the fits show that yield points described in both stress and strain spaces show linear hardening for this range of macroscopic strain norms. However, as for damage propagation, the slopes are found to be different for different load cases (Table 2); hardening in compression, tension and shear are considerably different. Hardening in compression is considerably larger than the rest, which is likely to be due to the fact that Drucker–Prager is used as the solid phase yield criterion, implying that the lack of hydrostatic compression yield may be partially upscaled to the macroscale, resulting in an increased evolution of the stress norm throughout the loading process. Although most models of trabecular bone at the macroscale use isotropic hardening (Garcia et al. 2009; Schwiedrzik et al. 2013) and nonlinear hardening laws (Garcia et al. 2009; Schwiedrzik and Zysset 2013), our results show a hardening behaviour which depends on the considered load case (i.e. tension, compression or shear) and a linear relationship between macroscopic yield stress/yield strain norm and macroscopic strain norm. However, the considered range of post-elastic strains is small in this study and hardening may become nonlinear if further loading is applied.

The validation of the results in this study is especially difficult to carry out. Damage evolution at the macroscale is usually evaluated by using cyclic loading experiments (Keaveny et al. 1994; Zysset and Curnier 1996). The configuration of these experiments is very different to the ones used in $\upmu$FE-related studies due to the BCs of the specimens. A similar problem occurs when validating the hardening results. Additionally, hardening in the literature is usually reported in the form of stress–strain curves, which indeed depend on the morphology of the specimens (high BV/TV specimens are likely to yield at larger stresses). Thus, samples of similar morphologies would be required to establish a meaningful comparison. Moreover, hardening values are usually reported for a larger range of strains, which are way beyond our small range of post-elastic strains. Nonetheless, we foresee that, in general, our hardening values are larger as they correspond to the inclined portion of the stress–strain curve immediately after yield.

The orthotropic assumption for the macroscopic elastic stiffness was used. The macroscopic strain is readily available, since it is directly applied through the considered BC (Wang et al. 2009). Nonetheless, in some shear load cases the homogenised stress presents some nonzero normal components (larger than one order of magnitude below the shear components of the stress tensor). This implies that even for these highly aligned samples, in-plane trabeculae experience normal stresses under macroscopic shear. It was shown by Sanyal et al. (2012) that macroscopic shear load cases are dominated by tensile solid phase stresses in trabeculae which are aligned $45^{\circ }$ from the shear in-plane axes. The homogenisation procedure is likely to have captured these tensile stresses when assessing the homogenised stress of the considered VE. This suggests that, at the macroscopic level, the normal and shear behaviours do have some interaction, which may outline a possible limit of the orthotropic macroscopic elastic assumption for trabecular bone.

Our study has a number of limitations. We use bovine trabecular bone specimens, whose results may not be readily comparable to human bone. Damage behaviour of bone at the tissue level has been researched in some previous studies (Schwiedrzik and Zysset 2013, 2015), but the maximum damage value is not known (Schwiedrzik and Zysset 2015) so we treated it as 90% reduction of the initial stiffness as an approximation and to avoid numerical difficulties related to the complete loss of stress carrying capacity; more research on the damage behaviour at the solid phase level is needed. We checked the effect of different thresholds (including 100% reduction) for one sample and found that the non-isotropic and loading mode dependency trend in damage is maintained and the difference between damage values at different macroscopic strain norms is small. There is plenty of experimental data on uniaxial load cases in literature (Keaveny et al. 1997; Bayraktar and Keaveny 2004; Sanyal et al. 2012; Manda et al. 2016), but these experiments do not permit the evaluation of the damaged orthotropic stiffness tensor. Additionally, these experiments do not allow for evaluation of samples submitted to different load cases, as yield or damage in one direction may affect the behaviour in other directions. Therefore, it may be argued that some of the results in this study are of higher order. Another limitation is the use of kinematic uniform BCs in the homogenisation procedure, which are known for being too stiff (Panyasantisuk et al. 2015), and may affect the damage morphology when compared to the relevant *in situ* case (Daszkiewicz et al. 2016). As in previous studies (Wolfram et al. 2012; Levrero-Florencio et al. 2016; Panyasantisuk et al. 2015), this study assumes the solid phase to be homogeneous. It has been shown that trabecular bone tissue has heterogeneous mineral density, and thus heterogeneous properties (Blanchard et al. 2013; Renders et al. 2008). However, the effects of mineral heterogeneities have minor influence on the apparent elastic properties of trabecular bone (Gross et al. 2012). Additionally, the effects of these heterogeneities in models with geometrical or material nonlinearities are still unknown and further research is needed to establish comparisons. The solid phase was modelled as a damage-plastic material without fracture, which is perhaps appropriate for the level of strains applied. It has been previously shown that ductile solid phase behaviour overestimates the experimental yield properties, especially at low BV/TV (Nawathe et al. 2013). Only strain-controlled uniaxial load cases for a relatively small number of samples were evaluated. In order to describe the full multiaxial behaviour of trabecular bone, more load cases are needed. However, the computational cost of performing a complete nonlinear simulation with the high resolution used in this study and with damage, plasticity, and an elastic homogenisation at each time increment is very high (it is important to point out that as damage grows, the stiffness matrix becomes increasingly unsymmetric, which decreases the convergence rate of the used iterative linear algebraic solver).

## Appendix: Implementation of the solid phase constitutive law

The mathematical operators are found in Section 2.2. The matrix representation of higher-order tensors is denoted with a bold upper-case character within square brackets, such as $[\mathbf{A }]$, and the vector representation of second-order tensors is denoted with a bold lower-case character within curly brackets, such as $\{\mathbf{a }\}$. However, note that $[\,]$ can also represent, in conjunction with $(\,)$, priority in the order of mathematical operations.

### Definition of the model

The constitutive model in this “Appendix” is based on the work of Schwiedrzik and Zysset (2013). In this study, we use a yield surface based on Schwiedrzik et al. (2013) with a damage cap based on Schwiedrzik and Zysset (2015).

The yield function is

10 $$\begin{aligned} f(\varvec{\sigma })=\sqrt{\varvec{\sigma }:{\mathbb {F}}:\varvec{\sigma }}+\mathbf{F }:\varvec{\sigma }-(1+H\,\overline{\varepsilon }^p)=0, \end{aligned}$$

where $\varvec{\sigma }$ is the Kirchhoff stress, and *H* is the hardening modulus (which is constant due to the assumption of linear isotropic hardening), and *D* is the damage variable, defined as

11 $$\begin{aligned} D=D_c(1-\text {e}^{-k_p\;\overline{\varepsilon }^p}), \end{aligned}$$

where $D_c$ and $k_p$ are positive constants related to damage evolution.

### Definition of the evolution equations

The residuals of the damaged quadric yield surface can be obtained by using an implicit time integration for the evolution equations. The closest-point projection method (CPPM) equations are defined as (note that the time increment subscripts are omitted for convenience)

12 $$\begin{aligned} \begin{Bmatrix} \mathbf{R }_{\varvec{\sigma }} \\ f \end{Bmatrix} = \begin{Bmatrix} \varvec{\sigma }-(1-D){\mathbb {D}}^e:(\varvec{\varepsilon }^{e\;\mathrm{trial}}-\varDelta \overline{\varepsilon }^p\,\frac{{\mathbf{N }}}{\Vert {\mathbf{N }}\Vert }) \\ \sqrt{\varvec{\sigma }:{\mathbb {F}}:\varvec{\sigma }}+{\mathbf{F }}:\varvec{\sigma }-(1+H\overline{\varepsilon }^p) \end{Bmatrix}, \end{aligned}$$

where $\mathbf{R }_{\varvec{\sigma }}$ is the residual of stresses, $\varvec{\varepsilon }^{e\;\mathrm{trial}}$ is the elastic trial strain, $\varDelta \overline{\varepsilon }^p$ is the increment of accumulated plastic strain, and ${\mathbf{N }}=\frac{\partial f}{\partial \varvec{\sigma }}=\frac{{\mathbb {F}}:\varvec{\sigma }}{\sqrt{\varvec{\sigma }:{\mathbb {F}}:\varvec{\sigma }}}+{\mathbf{F }}$. The residual of stresses can be converted to residual of elastic strains by using the term $(1-D)^{-1}({\mathbb {D}}^e)^{-1}$, so that

13 $$\begin{aligned} \begin{Bmatrix} \mathbf{R }_{\varvec{\varepsilon }^e} \\ f \end{Bmatrix} = \begin{Bmatrix} \varvec{\varepsilon }^e-\varvec{\varepsilon }^{e\;\mathrm{trial}}+\varDelta \overline{\varepsilon }^p\,\frac{{\mathbf{N }}}{\Vert {\mathbf{N }}\Vert } \\ \sqrt{\varvec{\sigma }:{\mathbb {F}}:\varvec{\sigma }}+{\mathbf{F }}:\varvec{\sigma }-[1+H(\overline{\varepsilon }^{p\;\mathrm{trial}}+\varDelta \overline{\varepsilon }^p)] \end{Bmatrix}, \end{aligned}$$

where $\overline{\varepsilon }^{p\;\mathrm{trial}}$ is the trial value of $\overline{\varepsilon }^p$. The solution variables are defined as

14 $$\begin{aligned} \mathbf{x }= \begin{Bmatrix} \varvec{\varepsilon }^e \\ \varDelta \overline{\varepsilon }^p \end{Bmatrix}. \end{aligned}$$

The residuals are linearised as

15 $$\begin{aligned}&\begin{Bmatrix} \text {d}\varvec{\varepsilon }^{e\;\mathrm{trial}} \\ 0 \end{Bmatrix}\nonumber \\&\quad = \begin{Bmatrix} \left[ {\mathbb {I}}+\varDelta \overline{\varepsilon }^p\frac{\partial }{\partial \varvec{\varepsilon }^e}\left( \frac{{\mathbf{N }}}{\Vert \mathbf{N }\Vert }\right) \right] :\text {d}\varvec{\varepsilon }^e+\text {d}\varDelta \overline{\varepsilon }^p\left[ \frac{{\mathbf{N }}}{\Vert \mathbf{N }\Vert }+\varDelta \overline{\varepsilon }^p\frac{\partial }{\partial \varDelta \overline{\varepsilon }^p}\left( \frac{{\mathbf{N }}}{\Vert \mathbf{N }\Vert }\right) \right] \\{} {\mathbf{N }}:\left( \frac{\partial \varvec{\sigma }}{\partial \varvec{\varepsilon }^e}:\text {d}\varvec{\varepsilon }^e+\frac{\partial \varvec{\sigma }}{\partial D}D'\text {d}\varDelta \overline{\varepsilon }^p\right) -H\text {d}\varDelta \overline{\varepsilon }^p \end{Bmatrix},\nonumber \\ \end{aligned}$$

where ${\mathbb {I}}_{ijkl}=\delta _{ik}\delta _{jl}$ and $\delta$ is the Kronecker delta. The increment in plastic strain, and the corresponding accumulated plastic strain, are defined as

16 $$\begin{aligned} \varDelta \varvec{\varepsilon }^p=\varDelta \overline{\varepsilon }^p\,\frac{{\mathbf{N }}}{\Vert {\mathbf{N }}\Vert } \end{aligned}$$

and

17 $$\begin{aligned} \overline{\varepsilon }^p=\overline{\varepsilon }^{p\;\mathrm{trial}}+\varDelta \gamma \,\Vert {\mathbf{N }}\Vert . \end{aligned}$$

After rearranging these terms, Eq. 15 becomes

18 $$\begin{aligned} \begin{Bmatrix} \text {d}\varvec{\varepsilon }^{e\;\mathrm{trial}} \\ 0 \end{Bmatrix} = [\mathbf{J }_{CPPM}] \begin{Bmatrix} \text {d}\varvec{\varepsilon }^e \\ \text {d}\varDelta \overline{\varepsilon }^p \end{Bmatrix}, \end{aligned}$$

where $[\mathbf{J }_{CPPM}]$ is the Jacobian of the CPPM scheme, defined as

19 $$\begin{aligned}{}[\mathbf{J }_{CPPM}]= \begin{bmatrix} {\mathbb {I}}+\varDelta \overline{\varepsilon }^p\frac{\partial }{\partial \varvec{\varepsilon }^e}\left( \frac{{\mathbf{N }}}{\Vert \mathbf{N }\Vert }\right)&\frac{{\mathbf{N }}}{\Vert \mathbf{N }\Vert }+\frac{\partial }{\partial \varDelta \overline{\varepsilon }^p}\left( \frac{{\mathbf{N }}}{\Vert {\mathbf{N }}\Vert }\right) \\{} (1-D){\mathbf{N }}:{\mathbb {D}}^e&{\mathbf{N }}:\frac{\partial \varvec{\sigma }}{\partial D}D'-H \\ \end{bmatrix}. \end{aligned}$$

The derivatives employed in the previous equation are

20 $$\begin{aligned} \begin{aligned}&\frac{\partial }{\partial \varvec{\varepsilon }^e}\left( \frac{{\mathbf{N }}}{\Vert {\mathbf{N }}\Vert }\right) =\frac{1-D}{\Vert {\mathbf{N }}\Vert }\left( {\mathbb {I}}-\frac{1}{(\Vert \mathbf{N }\Vert )^2}\mathbf{N }\otimes \mathbf{N }\right) :\frac{\partial \mathbf{N }}{\partial \varvec{\sigma }}:{\mathbb {D}}^e, \\&\frac{\partial \mathbf{N }}{\partial \varvec{\sigma }}=\frac{{\mathbb {F}}}{\sqrt{\varvec{\sigma }:{\mathbb {F}}:\varvec{\sigma }}}-\frac{({\mathbb {F}}:\varvec{\sigma })\otimes (\varvec{\sigma }:{\mathbb {F}})}{(\sqrt{\varvec{\sigma }:{\mathbb {F}}:\varvec{\sigma }})^3}, \\&\frac{\partial }{\partial \varDelta \overline{\varepsilon }^p}\left( \frac{\mathbf{N }}{\Vert \mathbf{N }\Vert }\right) =\mathbf 0 , \\&\frac{\partial \varvec{\sigma }}{\partial D}=-{\mathbb {D}}^e:\varvec{\varepsilon }^e, \end{aligned} \end{aligned}$$

and

21 $$\begin{aligned} D'=\frac{\partial D}{\partial \varDelta \overline{\varepsilon }^p}=D_c\,k_p\,\text {e}^{- k_p(\overline{\varepsilon }^{p\;\mathrm{trial}}+\varDelta \overline{\varepsilon }^p)}. \end{aligned}$$

### Consistent tangent operator

From the first row of Eq. 18, we obtain

22 $$\begin{aligned}&\left[ {\mathbb {I}}+\varDelta \overline{\varepsilon }^p\frac{1-D}{\Vert \mathbf{N }\Vert }\left( {\mathbb {I}}-\frac{1}{(\Vert \mathbf{N }\Vert )^2}\mathbf{N }\otimes \mathbf{N }\right) :\frac{\partial \mathbf{N }}{\partial \varvec{\sigma }}:{\mathbb {D}}^e\right] :\text {d}\varvec{\varepsilon }^e\nonumber \\&\quad +\,\frac{\mathbf{N }}{\Vert \mathbf{N }\Vert }\text {d}\varDelta \overline{\varepsilon }^p=\text {d}\varvec{\varepsilon }^{e\;\mathrm{trial}}. \end{aligned}$$

The first term of the LHS can be further developed by using

23 $$\begin{aligned} \text {d}\varvec{\varepsilon }^e=\frac{1}{1-D}({\mathbb {D}}^e)^{-1}: \left( \text {d}\varvec{\sigma }-\frac{\partial \varvec{\sigma }}{\partial \varDelta \overline{\varepsilon }^p}\text {d}\varDelta \overline{\varepsilon }^p\right) , \end{aligned}$$

such as

24 $$\begin{aligned} \begin{aligned}&\left[ {\mathbb {I}}+\varDelta \overline{\varepsilon }^p\frac{1-D}{\Vert \mathbf{N }\Vert }\left( {\mathbb {I}}-\frac{1}{(\Vert \mathbf{N }\Vert )^2}\mathbf{N }\otimes \mathbf{N }\right) :\frac{\partial \mathbf{N }}{\partial \varvec{\sigma }}:{\mathbb {D}}^e\right] :\text {d}\varvec{\varepsilon }^e\\&\quad =\left[ \frac{1}{1-D}({\mathbb {D}}^e)^{-1}+\varDelta \overline{\varepsilon }^p\frac{1}{\Vert \mathbf{N }\Vert }\left( {\mathbb {I}}-\frac{1}{(\Vert \mathbf{N }\Vert )^2}\mathbf{N }\otimes \mathbf{N }\right) :\frac{\partial \mathbf{N }}{\partial \varvec{\sigma }}\right] :\\&\qquad \left( \text {d}\varvec{\sigma }-\frac{\partial \varvec{\sigma }}{\partial \varDelta \overline{\varepsilon }^p}\text {d}\varDelta \overline{\varepsilon }^p\right) , \end{aligned} \end{aligned}$$

which, when applied to Eq. 22, becomes

25 $$\begin{aligned} \text {d}\varvec{\sigma }= & {} {\mathbb {P}}:\left( \text {d}\varvec{\varepsilon }^{e\;\mathrm{trial}}-\text {d}\varDelta \overline{\varepsilon }^p\frac{\mathbf{N }}{\Vert \mathbf{N }\Vert }\right) +\frac{\partial \varvec{\sigma }}{\partial \varDelta \overline{\varepsilon }^p}\text {d}\varDelta \overline{\varepsilon }^p\nonumber \\= & {} {\mathbb {P}}:\text {d}\varvec{\varepsilon }^{e\;\mathrm{trial}}+\left( \frac{\partial \varvec{\sigma }}{\partial \varDelta \overline{\varepsilon }^p}-{\mathbb {P}}:\frac{\mathbf{N }}{\Vert \mathbf{N }\Vert }\right) \text {d}\varDelta \overline{\varepsilon }^p, \end{aligned}$$

where the fourth-order tensor ${\mathbb {P}}$ is defined as

26 $$\begin{aligned} {\mathbb {P}}=\left[ \frac{1}{1-D}({\mathbb {D}}^e)^{-1}+\varDelta \overline{\varepsilon }^p\frac{1}{\Vert \mathbf{N }\Vert }\left( {\mathbb {I}}-\frac{1}{(\Vert \mathbf{N }\Vert )^2}\mathbf{N }\otimes \mathbf{N }\right) :\frac{\partial \mathbf{N }}{\partial \varvec{\sigma }}\right] ^{-1}. \end{aligned}$$

From the second row of Eq. 18, we obtain

27 $$\begin{aligned} \mathbf{N }:\text {d}\varvec{\sigma }+\left( \mathbf{N }:\frac{\partial \varvec{\sigma }}{\partial D}D'-H\right) \text {d}\varDelta \overline{\varepsilon }^p=0. \end{aligned}$$

By using Eq. 25 and Eq. 27, we obtain

28 $$\begin{aligned} \begin{aligned}&\mathbf{N }:\left[ \mathbb {P}:\text {d}\varvec{\varepsilon }^{e\;\mathrm{trial}}+\text {d}\varDelta \overline{\varepsilon }^p\left( -\mathbb {P}:\frac{\mathbf{N }}{\Vert \mathbf{N }\Vert }+\frac{\partial \varvec{\sigma }}{\partial \varDelta \overline{\varepsilon }^p}\right) \right] \\&\quad +\,\left( \mathbf{N }:\frac{\partial \varvec{\sigma }}{\partial \varDelta \overline{\varepsilon }^p}-H\right) \text {d}\varDelta \overline{\varepsilon }^p=0 \\&\quad \rightarrow \,\text {d}\varDelta \overline{\varepsilon }^p=\frac{\mathbf{N }:\mathbb {P}:\text {d}\varvec{\varepsilon }^{e\;\mathrm{trial}}}{\mathbf{N }:\left( \mathbb {P}:\frac{\mathbf{N }}{\Vert \mathbf{N }\Vert }-2\frac{\partial \varvec{\sigma }}{\partial \varDelta \overline{\varepsilon }^p}\right) +H}. \end{aligned} \end{aligned}$$

If this expression is inserted into Eq. 25, then

29 $$\begin{aligned} \text {d}\varvec{\sigma }=\mathbb {P}{:}\text {d}\varvec{\varepsilon }^{e\;\mathrm{trial}}+\frac{\mathbf{N }:\mathbb {P}:\text {d}\varvec{\varepsilon }^{e\;\mathrm{trial}}}{\mathbf{N }:\left( \mathbb {P}:\frac{\mathbf{N }}{\Vert \mathbf{N }\Vert }-2\frac{\partial \varvec{\sigma }}{\partial \varDelta \overline{\varepsilon }^p}\right) {+}H}\left( \frac{\partial \varvec{\sigma }}{\partial \varDelta \overline{\varepsilon }^p}-\mathbb {P}:\frac{\mathbf{N }}{\Vert \mathbf{N }\Vert }\right) . \end{aligned}$$

The final expression of the (generally unsymmetric) consistent elastoplastic tangent tensor, $\mathbb {D}^{ep}$, is obtained by taking into account that $\mathbb {D}^{ep}=\frac{\partial \varvec{\sigma }}{\partial \varvec{\varepsilon }^{e\;\mathrm{trial}}}$, such that

30 $$\begin{aligned} \mathbb {D}^{ep}=\;\mathbb {P}+\frac{\left( \frac{\partial \varvec{\sigma }}{\partial \varDelta \overline{\varepsilon }^p}-\mathbb {P}:\frac{\mathbf{N }}{\Vert \mathbf{N }\Vert }\right) \otimes (\mathbf{N }:\mathbb {P})}{\mathbf{N }:\left( \mathbb {P}:\frac{\mathbf{N }}{\Vert \mathbf{N }\Vert }-2\frac{\partial \varvec{\sigma }}{\partial \varDelta \overline{\varepsilon }^p}\right) +H}. \end{aligned}$$
